# The effect of preoperative stoma site marking on quality of life

**DOI:** 10.12669/pjms.341.14108

**Published:** 2018

**Authors:** Selda Karaveli Cakir, Turkan Ozbayir

**Affiliations:** 1 Selda Karaveli Cakir, RN, PhD, Assistant Professor, Nursing Department, Faculty of Health Science, Kastamonu University, Kastamonu, Turkey; 2 Turkan Ozbayir, RN, PhD, Associate Professor, Surgical Department, Faculty of Nursing, Ege University, Izmir, Turkey

**Keywords:** Quality of life, Stoma site marking, Surgical Stomas

## Abstract

**Objective:**

The objective of the study was to determine the effect of preoperative stoma site marking on the health- related quality of life (HRQOL).

**Methods:**

A nonrandomized, quasi-experimental design was used for the study performed from June 2013 to August 2014. The study sample (n:60) included patients for whom a stoma was opened after a planned colorectal surgery. The City of Hope Quality of Life-Ostomy Questionnaire (COHQOL-OQ) was used to measure HRQOL.

**Results:**

The mean age of the participants in the experimental group was 53.5±12.83, 18(60%) had colostomies, mean BMI was 25.46 ± 4.25 and mean age of that of the control group was 58.00 ± 14.22, 19(63.3%) had colostomies, mean BMI was 25.28 ± 5.00. A comparison of the two groups indicates that the sixth-month total score of the patients in the experimental group on (COHQOL-OQ) is higher than that of the control group (p<0.05).

**Conclusions:**

The study results demonstrated that patient who underwent stoma site marking reported higher HRQOL than those who did not.

## INTRODUCTION

Although surgical interventions aim to keep patients alive and increase the quality of their lives, these interventions may also cause new problems. One of the surgical interventions which alter persons' lifestyles and affect the quality of their lives is opening the intestinal stoma.^1^,^2^

When a stoma is opened, it profoundly changes the entire life of the individual deeply and requires adapting to a new lifestyle which is different from the one they are used to living until then. For a healthy individual, the elimination system is a physiological part of the body which they can control deliberately and independently. However, the stoma changes the function of the defecation, which makes the person dependent, deteriorates bodily integrity and leads to many physical and psychosocial problems.^1^-^5^ Patients with an open stoma are not only obliged to accept their diseases, but also organize their lives to accommodate the stoma.^6^

The anatomic location of the stoma is important for its care and the quality of life of the patient.^7^-^12^ Specialists claim that monitoring early and late complications, providing suitable care and solving underlying problems will improve these patients' quality of life. They also recommend marking the stoma site before the surgery.^9^-^11^

Quality of life scales are not sufficient for evaluating the quality of the lives of the patients with stoma.^13^,^14^ Certain scales specific for the disease and conditions should be used in order to measure the change made by the medical intervention more sensitively.^13^ In Turkey, researchers have used general quality of life scales to determine the quality of life of the patients with stoma.^3^,^15^ This study used the City of Hope Quality of Life-Ostomy Questionnaire COHQOL-OQ to evaluate the quality of life of the patients with stoma.^6^,^14^

The purpose of the study was to compare HRQOL in patients who received stoma site marking prior to surgery by a certificate wound, ostomy and continence nurse (CWOCN), to patients who did not receive preoperative marking.

## METHODS

A nonrandomized, quasi-experimental design was used for the study. Patients in the intervention group (n:30) received preoperative stoma marking by CWOCN and patients in the control group (n:30) did not. The study site was an 1800-bed University hospital where approximately 150 colostomy or ileostomy procedures are performed each year. The population of the study includes patients who had an open stoma and were treated in Ege University's Medical Faculty Hospitals' General Surgery Program between June 2013 and August 2014. The data were collected in face-to-face. The authors obtained written consent from Ege University's Medical Faculty Hospital's Clinical Research Ethics Committee before conducting the study (13-2/8). The aim of the study was explained to all patients through the Informed Voluntary Consent Form.

The requirements for patients to be included in the study sample were having an open stoma after a planned colorectal surgery, had no previous history of an abdominal stoma, being able to speak and understand Turkish, being willing to participate in the study and providing informed consent, being older than 18 years age, not having a diagnosed psychiatric illness, not having visual or hearing impairments, being literate, and accepting the six-months of post-operative monitoring. Patients who had an open stoma after an urgent surgery, experienced postoperative complications such as stoma necrosis, evisceration, retraction, scheduled for a stoma takedown within 6 months were not included in the study sample.

Multiple data were collected for this study including demographic information (age, sex, marital, educational and work status, type of stoma, time of stoma, disease, BMI), and functional lifestyle factors. Health-related quality of life measured using the City of Hope Quality of Life-Ostomy Questionnaire (COHQOL-OQ) which was created by Grant et al. 2003^16^ and translated into Turkish by Erol & Vural 2012. This scale is valid and reliable for Turkish society. COHQOL-OQ's reliability has an internal consistency of 0.95.^14^ This instrument contains 43 items. This is a Likert-type scale (0-10) including four sub-dimensions which are physical (items 1-11), psychological (items 12-24), social (items 25-36) and moral (items 37-43) sub-dimensions. Higher scores indicate a better quality of life.

The authors met the patients in the experimental (marked) and control (unmarked) groups the day before the operation and administered the patient identity form to them to obtain information about their socio-demographic status and health.^2^,^7^,^8^,^17^-^20^ Participant in the experimental group received standard preoperative marking by a WOCN following procedures outlined in the ASCRS and WOCN Joint Position Statement on the Value of Preoperative Stoma Marking for Patients Undergoing Fecal Ostomy Surgery.^21^ Then, their stoma sites were marked considering the anatomic evaluations and personal characteristics of the patients. After the operation, all patients received the same care provided by the stomal therapy nurse and the researcher. The quality of life of the participating patients after the operation were evaluated in the first and sixth months using the City of Hope Quality of Life-Ostomy Questionnaire (COHQOL-OQ) during this face-to-face interview.

The data were collected and analyzed using the Statistical Package for the Social Sciences software, version 20 (SPSS, Chicago, Illinois).^22^ Data were compared for equivalency and differences between groups using t-tests and analysis covariance. The p-values below 0.05 indicate that the difference between the two groups is significant.

## RESULTS

The study was conducted in 60 patients. The demographic data are presented in Table-I. The author did an analysis of the homogeneity of the groups and found that there was no statistically significant difference between the patients in the marked and unmarked groups in terms of introductory characteristics (age, gender, marital status, education status, employment status, type of stoma, opening stoma reason, BMI) (p>0.05).

**Table-I:** Demographic Data.

	Marked (n:30)	Unmarked (n:30)
Age, (mean)	53.5±12.83	58.00±14.22
*Sex [n (%)]*	
Female	13 (43.3%)	13 (43.3%)
Male	17 (56.6%)	17 (56.6%)
*Marital status [n (%)]*	
Single	7 (23.3%)	6 (20 %)
Married	23 (76.6 %)	24 (80 %)
*Educational status [n (%)]*	
High school diploma or less	14 (46.6%)	15 (50%)
More than diploma	16 (53.3%)	15 (50%)
*Work status [n (%)]*	
Working	5 (16.6%)	5 (16.6%)
Not working	25 (83.3%)	25 (83.3%)
*Type of stoma [n (%)]*	
Colostomy	18 (60 %)	19 (63.3%)
Ileostomy	12 (40%)	11 (36.6%)
*Time of stoma [n (%)]*	
Permanent	15 (50 %)	13 (43.3%)
Temporary	15 (50 %)	17 (56.6%)
*Opening stoma reason[n (%)]*	
Cancer	23 (76.6%)	26 (86.6%)
Non-cancer	7 (23.3%)	4 (13.3%)
BMI mean (SD)	25.46 ±4.25	25.28±5.00

**Fig. 1:**
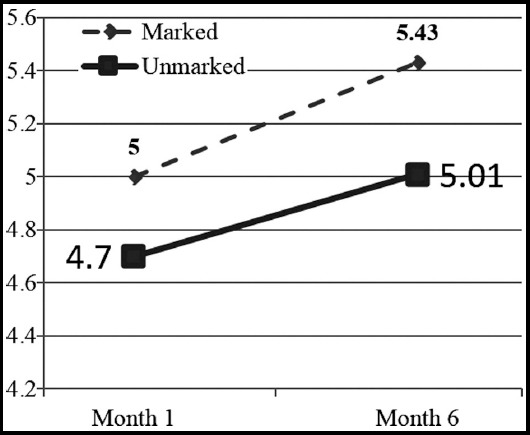
City of Hope Quality of Life-Ostomy Questionnaire Scores. At 6 months after ostomy surgery, subjects in the experimental (marked) group had significantly higher (p= .031) COHQOL-OQ scores than subjects in the control (unmarked) group.

Analysis of covariance, with age, gender, type of stoma as the covariant, was used to examine groups differences based on COHQOL-OQ scores. There was no statistically significant difference between the patients in the marked and unmarked groups (p>0.05). However, an increase in COHQOL-OQ at six month period following first month interval was significantly greater in the marked group than in the unmarked group (U=304; p=.031), indicating a significant increase in HRQOL among patients who received preoperative stoma marking. Fig.1 shows the COHQOL-OQ mean scores of the patients in the two groups in the first and sixth months after the operation.

Participants were asked about lifestyle factors that might influence HRQOL first month and six month after hospital discharge. Most participants (93% n=56) return to their own home, including 4 (7%) who lived alone. Group differences related stoma self-care functional lifestyle factors are displayed in Table-II. Although more patients in the marked group indicated higher levels of stoma self-management when compared to patients in the unmarked group, the differences were not statistically significant (p>0.05).

**Table-II:** Functional Lifestyle Factors at 6 Months Following Surgery.

Stoma functional lifestyle factors Self-care	Marked (n=30) Six month	Unmarked(n=30) Six month
**Empty ostomy pouch (χ^2^=, 4.63p=0.10)**		
Independent	67%(n=20)	50%(n=15)
With assistance	27%(n=8)	33%(n=10)
Unable	7%(n=2)	17%(n=5)
**Change ostomy pouching system(χ^2^=4.15, p=0.12)**		
Independent	60%(n=18)	53%(n=16)
With assistance	37%(n=11)	30%(n=9)
Unable	3%(n=1)	17%(n=5)

## DISCUSSION

We found that preoperative stoma site marking leads to a significantly better quality of life. This findings adds to a growing body of evidence that preoperative stoma marking enhances postoperative HRQOL. Our findings are similar to those reported by Mahjoubi et al., Pearson et al., and Mydick and McKenna et al. who reported that patients receiving preoperative stoma site marking significantly higher HRQOL scores than unmarked patients.^7^,^8^,^12^ The majority of our study participants underwent ostomy surgery due to a cancer diagnosis; therefore, the cancer diagnosis itself may have impacted HRQOL. Having a diagnosis of cancer can be an additional stressor for ostomy patients.^8^

Smith et al.^23^ reported that patients who had permanent stomas had better quality of life than patients whose stomas were temporary, suggesting that adjustment to a permanent disability is easier and faster, despite the fact that this medical situation is objectively worse. Yau et al.^24^ conducted a study with 186 patients and found that the stoma being permanent or temporary had an important influence on patients' quality of life. In this study, we used a validated COHQOL-OQ questionnaire, and it suggested that such a difference does not exist. The main reason for the differences in the quality of life of the two groups was not the type of stoma but whether its site was preoperatively marked as an independent factor.

Lifestyle functions related to HRQOL can also be impacted by stoma location. Sun et al.,^25^ found that many individuals with ostomies faced challenges related to bowel function, activity limitations, and clothing restrictions due to the placement of the stoma. Their findings suggest that a poorly located ostomy required more diligent constant monitoring to avoid leakage and embarrassing accidents, which participants found emotionally exhausting and stressful.

### Limitations

This study is limited to patients who had a planned post-colorectal surgery stoma opening. The study was conducted in a single institution. Finally we acknowledge that the influence of a cancer diagnosis leading to a fecal ostomy and HRQOL.

## CONCLUSION

Stoma site marking increase HRQOL during the postoperative period. Based on results of this study, we suggest that CWOCN marked stoma site prior to ostomy surgery. Preoperative stoma site marking builds a close relationship between the patients and stomal therapy nurses and helps the post-operative training to be more effective. Patients who can do their own stoma care can adapt to the stoma, and their quality of life is enhanced.

### Authors' Contribution

**SKC:** Conception and design, data collection, drafting, statistical analysis manuscript writing.

**TO:** Conception, design and review of literature.

## References

[1] Yasan A, Unal S, Gedik E, Girgin S (2008). Quality of life, depression and anxiety among patients who haveundergone permanent or temporary ostomy. Anatol J Psychiatry.

[2] De la Quintana Jimenez P, Pastor Juan C, Prados Herrero I, Perez Lopez C, Gonzalez Fuentes M, de Mena Casaseca C (2010). A Prospective, Longitudinal, Multicenter, Cohort Quality-of-Life Evaluation of an Intensive Follow-Up Program for Patients with a Stoma. Ostomy Wound Manag.

[3] Tari O (2011). Abdominal Stomali Hastalarda Yaşam Kalitesinin İncelenmesi. (Analyzing Life Quality of Abdominal Stoma of the Patients.). Master's thesis.

[4] Zajac O, Spychała A, Murawa D, Wasiewicz J, Foltyn P, Połom K (2008). Quality of Life Assessment in Patients with a Stoma Due to Rectal Cancer. Rep Pract Oncol Radiother.

[5] Popek S, Grant M, Gemmill R, Wendel CS, Mohler MJ, Rawl SM (2010). Overcoming Challenges: Life with an Ostomy. Am J Surg.

[6] Arguden Y (2008). Quality of Life. Once Kalite Derg.

[7] Mahjoubi B, Goodarzi KK, Mohammad-Sadeghi H (2010). Quality of Life in Stoma Patients: Appropriate and Inappropriate Stoma Sites. World J Surg.

[8] Person B, Ifargan R, Lachter J, Duek SD, Kluger Y, Assalia A (2012). The Impact of Preoperative Stoma Site Marking on the Incidence of Complications, Quality of Life, and Patient's Independence. Dis Colon Rectum.

[9] Colwell JC, Gray M (2007). Does Preoperative Teaching and Stoma Site Marking Affect Surgical Outcomes in Patients Undergoing Ostomy Surgery?. J Wound Ostomy Continence Nurs.

[10] Registered Nurses'Association of Ontario (2009). Ostomy Care and Management, Toronto, Canada.

[11] (2010). Wound Ostomy and Continence Nurses Society (WOCN). Management of the Patient with a Fecal Ostomy: Best Practice Guideline for Clinicians. J Wound Ostomy Continence Nurs.

[12] Maydick D (2016). A Descriptive Study Assessing Quality of Life for Adults With a Permanent Ostomy and the Influence of Preoperative Stoma Site Marking. Ostomy Wound Manage.

[13] Eser E (2012). Basis of Conceptual Health Related Quality of Life. Cerrahi Bakim ve Yaşam Kalitesi Sempozyumu.

[14] Erol F, Vural F (2012). Validity and Reliability of The City of Hope Quality of Life Ostomy -Scale for The Turkish Patients With Ostomy. J Res Develop Nurs.

[15] Altuntas YE, Kement M, Gezen C, Eker HH, Aydin H, Sahin F (2012). The Role of Group Education on Quality of Life in Patients with a Stoma. Eur J Cancer Care.

[16] Grant M, Ferrel B, Dean G, Uman G (2004). Revision and psychometric testing of the city of hope quality of life – ostomy questionnaire. Qual Life Res.

[17] Marguis P, Marrel A, Jambon B (2003). Quality of Life in Patients with Stomas: The Montreux Study. Ostomy Wound Manage.

[18] Ma N, Harvey J, Stewart J, Andrews L, Hill AG (2007). The Effect of Age on The Quality of Life of Patients Living with Stomas: A Pilot Study. ANZ J Surg.

[19] Toth PE, Thompson SJ, Davis JS (2012). Factors Impacting the Quality of Life of People with an Ostomy in North America. J Wound Ostomy Continence Nurs.

[20] McKenna LS, Taggart E, Stoelting J, Kirkbride G, Forbes GB (2016). The Impact of Preoperative Stoma Marking on Health-Related Quality of Life: A Comparison Cohort Study. J Wound Ostomy Continence Nurs.

[21] (2007). American Society of Colon and Rectal Surgeons Committee Members, Wound Ostomy Continence Nurses Society Committee Members. ASCRS and WOCN joint position statement on the value of the pre-opertative stoma marking for patients undergoing fecal ostomy surgery. J Wound Ostomy Continence Nurs.

[22] SPSS Inc (2011). SPSS Statistics for Windows, Version 20.0.

[23] Smith DM, Loewenstein G, Jankovic A, Ubel PA (2009). Happily Hopeless: Adaptation to Permanent, but not to a Temporary, Disability. Health Psychol.

[24] Yau T, Watkins D, Cunningham D, Barbachano Y, Chau I, Chong G (2009). Longitudinal Assessment of Quality of Life in Rectal Cancer Patients with or without Stomas Following Primary Resection. Dis Colon Rectum.

[25] Sun V, Grant M, McMullen CK, Altschuler A, Mohler MJ, Hornbrook MC (2013). Surviving colorectal cancer: long-term, persistent ostomy-specific concerns and adaptations. J Wound Ostomy Continence Nurs.

